# Supplementary material for: Identification of photoperiod-induced specific miRNAs in the adrenal glands of Sunite sheep (Ovis aries)
Source: Front Vet Sci. 2022 Jul 22;9:888207. doi: 10.3389/fvets.2022.888207 (PMC9354845; doi:10.3389/fvets.2022.888207)
Supplement: Supplementary file 1 [file Data_Sheet_1.ZIP › Supplementary file/miRDeep2 file.zip!/miRDeep2 file/Know_Structure/LP42A3/oar-mir-323b.pdf]

miRBase precursor : oar-mir-323b
Total read count : 14
oar-miR-323b read count : 14
remaining reads : 0

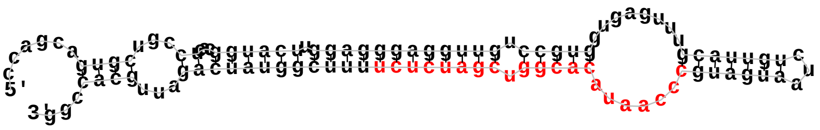

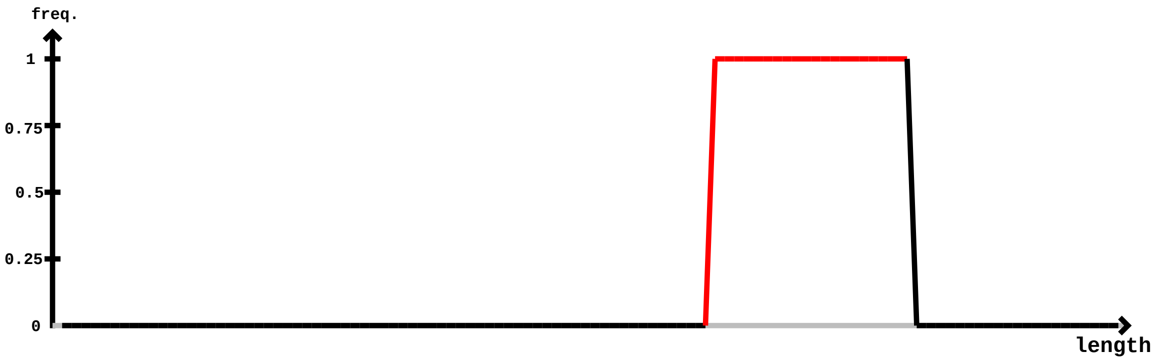

oar-miR-323b

```
5'- ccagcagugcugccucacgguacuuggagggagguuguccguggugaguuugcauugucuaaugaugcccaauacacggucgaucucuuuucgguaucagauugcaccgg   -3'    exp
    ......((((...((...((((((.((((((((((((.(((((........(((((((...)))))))......))))).))))))))))))))))))))...))))...    reads      mm          sample
    ...................................................................cccaauacacggucgaucucu......................    14         0           seq
```
